# Using the CustusX toolkit to create an image guided bronchoscopy application: Fraxinus

**DOI:** 10.1371/journal.pone.0211772

**Published:** 2019-02-08

**Authors:** Janne Beate Lervik Bakeng, Erlend Fagertun Hofstad, Ole Vegard Solberg, Jon Eiesland, Geir Arne Tangen, Tore Amundsen, Thomas Langø, Ingerid Reinertsen, Tormod Selbekk, Håkon Olav Leira

**Affiliations:** 1 Research Group for Medical Technology, Department of Health, SINTEF Technology and Society, Trondheim, Norway; 2 Department of Thoracic Medicine, St. Olavs hospital, Trondheim, Norway; 3 Department of Circulation and Medical Imaging, Faculty of Medicine, Norwegian University of Science and Technology, Trondheim, Norway; 4 Norwegian National Advisory Unit for Ultrasound and Image-Guided Therapy, St. Olavs hospital, Trondheim, Norway; Harvard University, UNITED STATES

## Abstract

**Purpose:**

The aim of this paper is to show how a specialized planning and guidance application called Fraxinus, can be built on top of the CustusX platform (www.custusx.org), which is an open source image-guided intervention software platform. Fraxinus has been customized to meet the clinical needs in navigated bronchoscopy.

**Methods:**

The application requirements for Fraxinus were defined in close collaboration between research scientists, software developers and clinicians (pulmonologists), and built on top of CustusX. Its superbuild system downloads specific versions of the required libraries and builds them for the application in question, including the selected plugins. New functionality is easily added through the plugin framework. The build process enables the creation of specialized applications, adding additional documentation and custom configurations. The toolkit’s libraries offer building blocks for image-guided applications. An iterative development process was applied, where the clinicians would test and provide feedback during the entire process.

**Results:**

Fraxinus has been developed and is released as an open source planning and guidance application built on top of CustusX. It is highly specialized for bronchoscopy. The proposed workflow is adapted to the different steps in this procedure. The user interface of CustusX has been modified to enhance information, quality assurance and user friendliness with the intention to increase the overall yield for the patient. As the workflow of the procedure is relatively constant, some actions are predicted and automatically performed by the application, according to the requirements from the clinicians.

**Conclusions:**

The CustusX platform facilitates development of new and specialized applications. The toolkit supports the process and makes important extension and injection points available for customization.

## Introduction

Bronchoscopy is the endobronchial procedure for inspection and diagnostic sampling in the airways, e.g. to diagnose lung cancer. Navigating the flexible video bronchoscope in the lungs is difficult due to the numerous divisions in the tree structure of the airways. Another challenge is the lack of direct visibility of smaller peripheral lesions located outside the airways. This makes it difficult to hit the target for the diagnostic sampling using biopsy or fine needle aspiration. Even with fluoroscopy guidance, the diagnostic success rate for bronchoscopically non-visible tumors is as low as 15% compared to 80% for visible tumors, dependent on tumor size, the doctors experience and the method used for sampling [1–3].

Navigated bronchoscopy represents a possibility to reduce these challenges and thus increase the biopsy success rate. Commercially available systems like Superdimension Navigation system (Medtronic, USA) and Veran SpinDrive (Veran Medical Technologies, Inc, St. Louis, USA) provide navigation based on electromagnetic tracking (EMT) of the bronchoscopic tools. Systems like Cybernet DirectPath^®^ (Cybernet Systems Co, Ltd., Tokyo, Japan) and LungPoint (Broncus Medical Inc., San Jose, USA) offer no navigation possibilities, but have more advanced visualization of e.g. the airways and the tumor based on computer tomography (CT) images. However, all the commercially available systems are associated with costs related to purchase and use (consumables), which both can be considerable. This limits the widespread use of these systems in clinics. A more detailed description of the existing systems for navigated bronchoscopy, both commercial and research, are presented by Reynisson et al. [4].

We have developed a system, Fraxinus (www.custusx.org/fraxinus), which is made freely available in both code and binary form, as is becoming the norm in science [5]. The system is customized to meet the clinical needs for both procedural planning and guidance of bronchoscopy. A goal for the application is that it should run on off-the-shelf computers, which will make the technology available without additional investments in hardware or software. This is specially important for the use in smaller hospitals and in developing countries. Without a planning system, the procedural preparations involves inspection of the CT images in 2D orthogonal slices, which is difficult to interpret for 3D navigation with the bronchoscope. Fraxinus will provide more advanced visualizations based on CT images, to be used both in the planning phase and during the procedure. The system will find the optimal route to a target position (e.g. a tumor) and provide virtual bronchoscopy with different visualization possibilities. Fraxinus is based on CustusX [6], which is a software platform for image-guided interventions.

In 2015, CustusX was released as an open source research platform with a focus on intraoperative navigation and ultrasound (US) imaging. It has been continuously developed, tested and adapted for more than 15 years in a multidisciplinary cooperation between clinical and technological researchers.

The platform provides a toolkit with features that are aimed at facilitating the development of new software applications. It offers ways of adding and modifying features, customizing the build process, creating installers, and adding quality assurance in the form of testing and continuous integration.

The main goal of this paper is to demonstrate the feasibility of developing an open source software application using the CustusX toolkit. We will also demonstrate the usefulness of the developed application, an easily applicable planning and guidance system for bronchoscopy. The focus is on how the CustusX toolkit is applied along with implementation of new features when creating this specialized application. Optimization through an intuitive user-interface and identifying the needed features for the procedure was ensured in close collaboration with pulmonologists by mirroring the clinical workflow in detail, presenting context sensitive guidance, displaying useful widgets and providing automatic system state transitions.

The following two chapters describe the system specifications used for guiding the software development and how these requirements are implemented using the CustusX toolkit. Then we present how Fraxinus performs with patient CT data, before discussion and conclusion wraps up the paper.

## System specifications

The application specifications were defined over many years through an iterative process involving both technical and clinical personnel. In order to design a system that meets the clinical requirements, it is critically important for technical personnel to understand the tasks and challenges faced by the clinicians before and during the procedure. To gain first-hand experience with the procedure, the software developers therefore attended bronchoscopy procedures in the bronchoscopy suite where CustusX was used [7, 8]. The system specifications were then further refined through numerous discussions and demonstrations of early versions of the software where all team members contributed to the process. This resulted in the specification of the required features and the workflow.

Specializing CustusX to fit these needs involved:

- *Adding* state-of-the-art algorithms for extraction of anatomic structures like vessels, airways and the center lines of the airways [9, 10].
- *Hiding* unnecessary features from the GUI.
- *Optimizing* the GUI and visualizations to fit the workflow specified.
- *Automating* actions that can be predicted based on the procedure and available data.

### User interface

The design of the GUI in Fraxinus remained similar to the GUI in CustusX. Key concepts like a main window based on the Qt QMainWindow class, which provides menus, toolbars, configurable widgets and a central view area, were kept relatively untouched. How the user interacts with the system, using a mouse and keyboard, was also kept the same.

The main differences between the Fraxinus GUI and the CustusX GUI lie in the contents available to the user. In Fraxinus, many unnecessary and advanced features are not shown to the user. This provides an optimized and uncluttered GUI, better suited to the clinician’s workflow in the bronchoscopy suite. The user will be presented with the most appropriate tools, layout and help for each consecutive steps in the workflow.

### Features and workflow

In CustusX, the clinical procedure is modeled as a workflow divided into a sequence of steps. This concept is preserved, but adjusted to fit the specifications for Fraxinus, i.e. for bronchoscopy planning and guidance.

**Fig 1:**
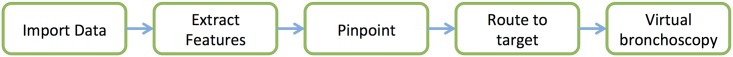
Bronchoscopy workflow as implemented in Fraxinus. Patient data is imported, then processed in several stages until virtual bronchoscopy can commence.

A specific workflow for a bronchoscopy intervention was identified, see Fig 1. The intention is that the user goes through these step subsequently, as each step depends on some input from the preceding steps. The identified necessary features for Fraxinus is included in the different steps of the workflow described below.

#### Import

Patient images (e.g. CT) are imported by selecting a Digital Imaging and Communications in Medicine (DICOM) folder. The DICOM folder might contain several data sets, and the user selects the relevant images to load into the application. Now that the images are available to be visualized, they can be inspected and their properties can be adjusted.

#### Extract features

The volume formed by the patients images can be processed in order to extract structures of interest. An algorithm for airway segmentation is available at this stage. The volume is automatically reconstructed from a set of images, and then the algorithm automatically extracts the centerlines and generates a surface segmentation of the lung airways. The airway segmentation algorithm is based on the work described in [9, 10]. It is a fast and automatic method for extracting airway structures from different image modalities (CT, MR and US) by utilizing the computational power of graphic processing units (GPUs). The airway segmentation algorithm can also be run on the central processing unit (CPU) at the cost of longer processing time. When the process is completed, the resulting structures can be visualized together with the input images to verify that the algorithm has produced a satisfactory result.

#### Pinpoint

A point of interest, such as a suspicious lesion in the lungs, can be identified and marked. Inspection and pinpointing can be performed in the 2D or 3D views of this workflow step. The point can be resampled, named, moved, deleted, colored and visualized together with the other structures extracted from the patient’s images. At this point the user is able to get an overview of the relative placements of the structures of interest that should be examined during the bronchoscopy.

#### Route-to-target

The two previous steps generated a map of the lung airways and a target of interest. By combining this information with an algorithm called *route-to-target*, we are able to find a path for the bronchoscope to move from the point of entry to the specific target. The *route-to-target* algorithm calculates a route along the centerline of the lung airways that will guide the user to the point inside the bronchi that is closest to the target. First, all branches of the airway tree are automatically identified and connected. Thus, each branch object contains information about positions along the branch, and the parent and child branches it is connected to. All branches but the trachea (windpipe) have one parent branch. Second, the path from the target to the top of the trachea is computed. The centerline position closest to the target is selected, and all positions to the top of the corresponding branch are included in the path. Furthermore, all positions of the parent branch are included. This procedure is repeated until the top of the trachea is reached. The route-to-target is then defined by all the points included in this path sorted in the opposite order. This step can be repeated by redefining the target point.

#### Virtual bronchoscopy

Virtual bronchoscopy (VB) allows the user to experience and plan the intervention before starting the clinical procedure. VB allows traveling through the lung airways from the top of the trachea, through different bronchi, all the way to the target. The movement is restricted to the centerlines of the tubular structures created by the airway segmentation algorithm.

In Fraxinus, we propose two different modes of VB: (I) In *fly-through* mode the user will experience traveling through the lung tree as if looking at video from a bronchoscope. The airways for VB are created by smoothing the centerlines from the segmented airways, and drawing artificial tubes around them where the diameter of the tubes is set by the anatomical location of the tube, i.e. trachea is largest. These synthetic airways were created after the clinicians experienced that the the original extracted airways did not provide sufficient resolution in the periferal (smaller) branches. (II) The novel VB method *cut-planes* was created to show information in the CT images not visible in the *fly-through* mode, like tumors and blood vessels. Updated *cut-planes* are created and applied continuously to the structures in the 3D view. The cut-planes is set to cut along the axes of the current position on the centerline. The camera is set to see the entire thorax from an angled and elevated position. The movement and control options are the same as for the fly-through mode, but the user can inspect surrounding structures when moving through the lung tree.

### Software features and automation

Many of the features requested in Fraxinus were already available in CustusX, like saving/loading, import image data, visualization among other. Missing features were developed and either added to the Fraxinus open source code, or included in the CustusX open source platform. Specifically, there are several tailored widgets made specially for Fraxinus which is not added to CustusX as they are deemed too procedure specific.

Fraxinus defines a set of automatic actions related to its workflow and some of its algorithms. If the actions performed by the users can be recognized as part of a workflow sequence, the subsequent steps of the procedure can be automated by the software. By monitoring how the user interacts with the system at various stages in the procedure it was possible to identify and implement actions to be automated. One such example is automatically visualizing an imported data set in the 3D view.

A user manual has been added to the application specific help system. It describes the intended way to use Fraxinus, details about each of the workflow stages, known limitation and troubleshooting. The CustusX help system is at the base of this manual which provides more generic help and instructions that the user might find useful.

## Software development

The CustusX platform uses the plugin-centered architecture from CTK (The Common Toolkit) [11] making it a flexible platform for building new applications. The CTK plugin framework is based on OSGi (Open Services Gateway initiative) [12]. Plugins can be used to extend or alter the core system behaviour through extension point services. Developers can use the libraries of the platform as building blocks for assembling their own customized application. The graphical user interface can be customized to fit the applications needs. As CustusX is not specialized towards any particular procedure, the amount of options available might become distracting to a specialized user. Additionally, the generic workflow might not sufficiently cover the clinical procedure at hand.

**Fig 2:**
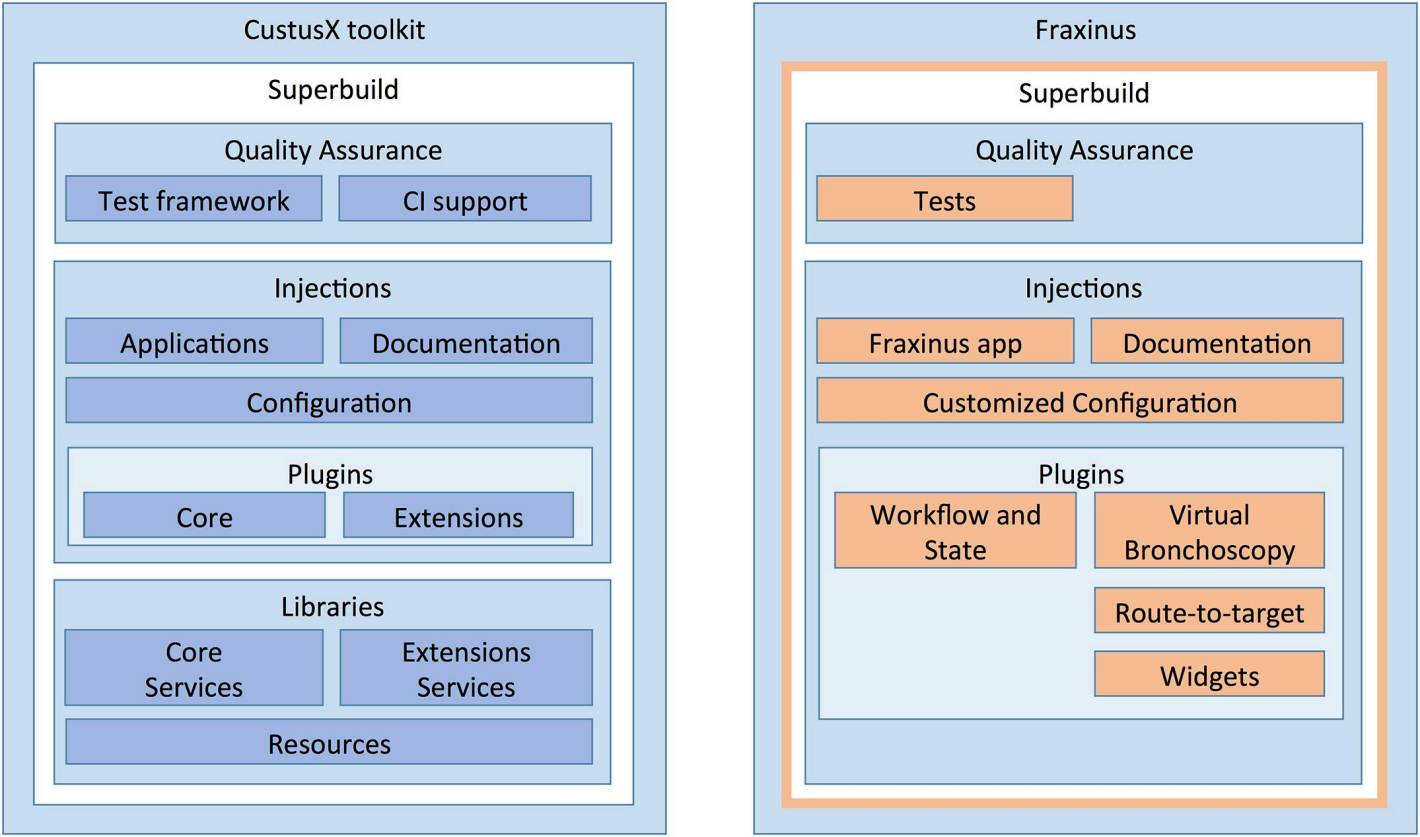
CustusX toolkit expansion points (left) and Fraxinus expansions (right). Light blue boxes show possible expansion points. Orange boxes indicates where Fraxinus-specific features have been added.

The Fraxinus software development process is performed in iterations allowing for corrections along the way, thus following an agile process [13]. It involves programming, testing, bug fixing and documentation of the software. The CustusX toolkit facilitates these tasks. It offers many expansion points for developing new applications. Fig 2 (left) shows an overview of these expansion points. The superbuild administers the build process. The continuous integration (CI) tools add tests and automate them. At certain points you can add code into the build process to modify its behaviour. Plugins allow features to be added or modified. The libraries adds building blocks.

Fraxinus is implemented as a set of plugins that connect to CustusX expansion points. These extensions can either add, modify or remove functionality. As Fig 2 (right) shows, Fraxinus utilizes many of the possibilities of the toolkit. It adds a thin layer to the superbuild system, adds tests and code to build a specialized application with documentation and configuration files, it replaces a core plugin, and adds several extension plugins.

### Superbuild

In CustusX the superbuild system [6] defines and maintains a set of components which CustusX depends on. These components are either external libraries or plugins. The superbuilds responsibility is to download, configure and build all components that CustusX requires. For example, upgrading the version of a library involves changes to the superbuild code by pointing at the right version in its repository.

Fraxinus adds a layer to the superbuild system by defining new superbuild components. One component is the Fraxinus application itself, which is injected into the build through an injection point in the build system. The other components Fraxinus adds are plugins.

### Quality assurance

Each library or plugin in CustusX is accompanied by a set of tests, either unit or integration tests. When building a library or plugin most will have a parallel test library. The superbuild system is responsible for collecting and registering all test libraries to the central test executable. This executable, containing all the tests, can be run either locally or be set up to run by a CI system like Bamboo (Atlassian). (https://www.atlassian.com/software/bamboo).

Fraxinus tests are added to the test suite and are made available together with CustusX’s tests. Using the tagging system from Catch (https://github.com/catchorg/Catch2), a subset of all test can be run by specifying the desired tags. Fraxinus is tested regularly on a Bamboo system setup where unit tests and integration tests are triggered on every git commit and once nightly. The Bamboo integration was adopted unchanged from CustusX.

### Build process modification points

To be able to customize the build process the CustusX toolkit offers injections points into the build process. They are CMake [14] macros or functions for adding custom applications, plugins, documentation, configuration files or options.

To create the Fraxinus application a small plugin was added into CustusX which builds the executable and links in all appropriate resources. This was done by creating a main function, which creates an application loop based on Qt (http://www.qt.io), loads a resource pack for custom icons, creates a CustusX main window and initializes the logic from CustusX that makes the platform available. It also adds custom configuration files and documentation which is distributed with the Fraxinus installation. By default, the CustusX application is built by the superbuild system, but it is turned off when building Fraxinus. Other modified or added functionality in Fraxinus is added as plugins.

### Plugins

CustusX uses the CTK Plugin Framework [11], which adds a central hub for handling plugins. A plugin must have an activator which registers itself to the hub as the shared library is loaded. Optionally it also supplies an implementation of one or more service extension points. The shared library can be loaded and unloaded at runtime by interacting with the hub through a widget inside CustusX.

Fraxinus adds four plugins. One plugin implements a StateService, another a FilterService and two implements a GUIExtenderService. *Workflow and state plugin* modifies the workflow state machine by defining the states corresponding to the steps in the bronchoscopy procedure. It is a core plugin and replaces the matching core plugin from CustusX. Next are the extension plugins: *Route-to-target* which adds functionality for navigating through the lung tree towards a target. *Virtual Bronchoscopy* include GUI for maneuvering along the centerline of the lung airways. *Widgets plugin* adds custom widgets which offer other specialized user interfaces tailor-made based on the requirements from the clinicians.

### Libraries

The building blocks of CustusX are its resource libraries, which depends on many external libraries as VTK(http://vtk.org), ITK(http://itk.org), CTK, Qt, OpenIGTLink [15], Eigen(http://eigen.tuxfamily.org), OpenCV [16] and others. They contain C++ components suitable for building image-guided intervention software.

Fraxinus utilizes many of the resource libraries from CustusX to build its plugins. It does not add any libraries of its own, although technically that would be feasible.

## System demonstration

Information on how to aquire Fraxinus can be found on the Fraxinus website (https://www.custusx.org/fraxinus). There is a short introduction video presenting the application, links to downloadable binaries, datasets which can be used for testing, and also links to the source code.

The intended audience for the binary application is medical researchers who perform endobronchial procedures for inspection and diagnostic sampling in the airways of humans. The source code is made available for anyone interested in the underlying mechanics and it is possible for anyone to modify and build their own version based on the Fraxinus source code.

### Source code

The source code for Fraxinus is written mostly in C++ and it is available as an open source git repository on GitHub (https://github.com/SINTEFMedtek/Fraxinus). This is a relatively small respository as most of the code behind Fraxinus is shared with CustusX. GitHub (https://github.com/) is a development platform for hosting and reviewing code, managing projects and building software and git (https://git-scm.com/) is a distributed version control system.

Fraxinus uses the same superbuild system as CustusX which is based on a python and CMake integration. Before trying to run the superbuild it is important that the prerequisites are met. This information can be found under developer documentation on the Fraxinus website. Running the script should make sure valid versions of all dependent libraries are acquired and built appropriately.

### Application

Fraxinus is available to download for Windows, Ubuntu and Mac. The application is packed in an installer which will take care of installing all the necessary files needed for the application to run.

Running the application for the first time will automatically create a folder, Fraxinus_settings, containing system logs, settings and preferences. Deleting this folder will reset the system to it factory state.

As a user, first create a new patient, giving the application a place to save all patient specific data. Then, import a DICOM set of CT images which will be used for the virtual bronchoscopy (VB). After this, the application guides the user through the workflow steps leading up to the VB simulation.

Furthur documentation on how to use the application is available on the website custusx.com/fraxinus. The short introduction video shows the normal flow of a procedure and there is also a link to written user documentation.

### Fraxinus demonstration

Fraxinus was tested running on a laptop computer (Intel^®^ Core^™^ i7-7700HQ CPU @ 2.80GHz, 16 GB memory, ASUS, Windows 10, NVIDIA GeForce GTX 1050 Ti). An example CT scan of a patient was used for this demonstration. The CT images were acquired using a thorax lung scan protocol with 620 slices of 512x512 pixels, element spacing 0.676x0.676x0.5 mm, and slice thickness 1.0 mm. Use of the patient CT data was approved by the Regional Committees for Medical and Health Research Ethics (REC) in Norway, approval number: 2015/1913/REK sør-øst C. A written consent was obtained for each included patient (anonymized data) in the available database, which can be found at https://datadryad.org/resource/doi:10.5061/dryad.mj76c. Thorax CT patient 015 was used for the images in this manuscript.

**Fig 3:**
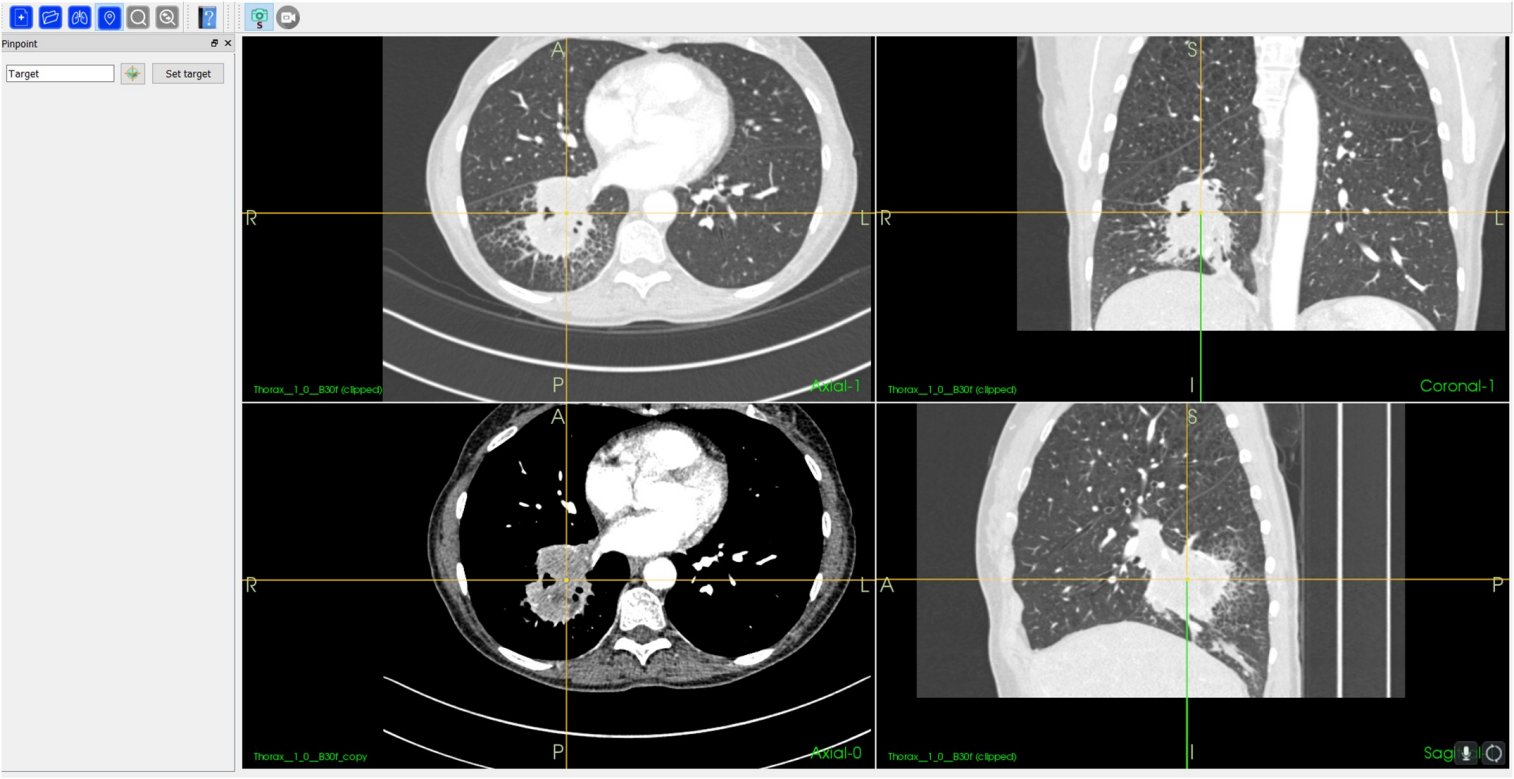
Screenshot of the pinpoint workflow step where a target for the VB is selected.

The DICOM CT data was saved on a solid state drive (SSD) and was successfully imported into the application. The airways and centerline algorithm extracting structures from the lungs, ran on the GPU. This process took 19.89 seconds, the most time-consuming step of the application. All other steps were processed in less than 1 second. A selected position for tissue sampling was pinpointed as a target. Fig 3 shows how the target position can be found and adjusted in axial, sagittal and coronal slices from the CT. The optimal route-to-target was found based on the target position and the centerline of the airways.

**Fig 4:**
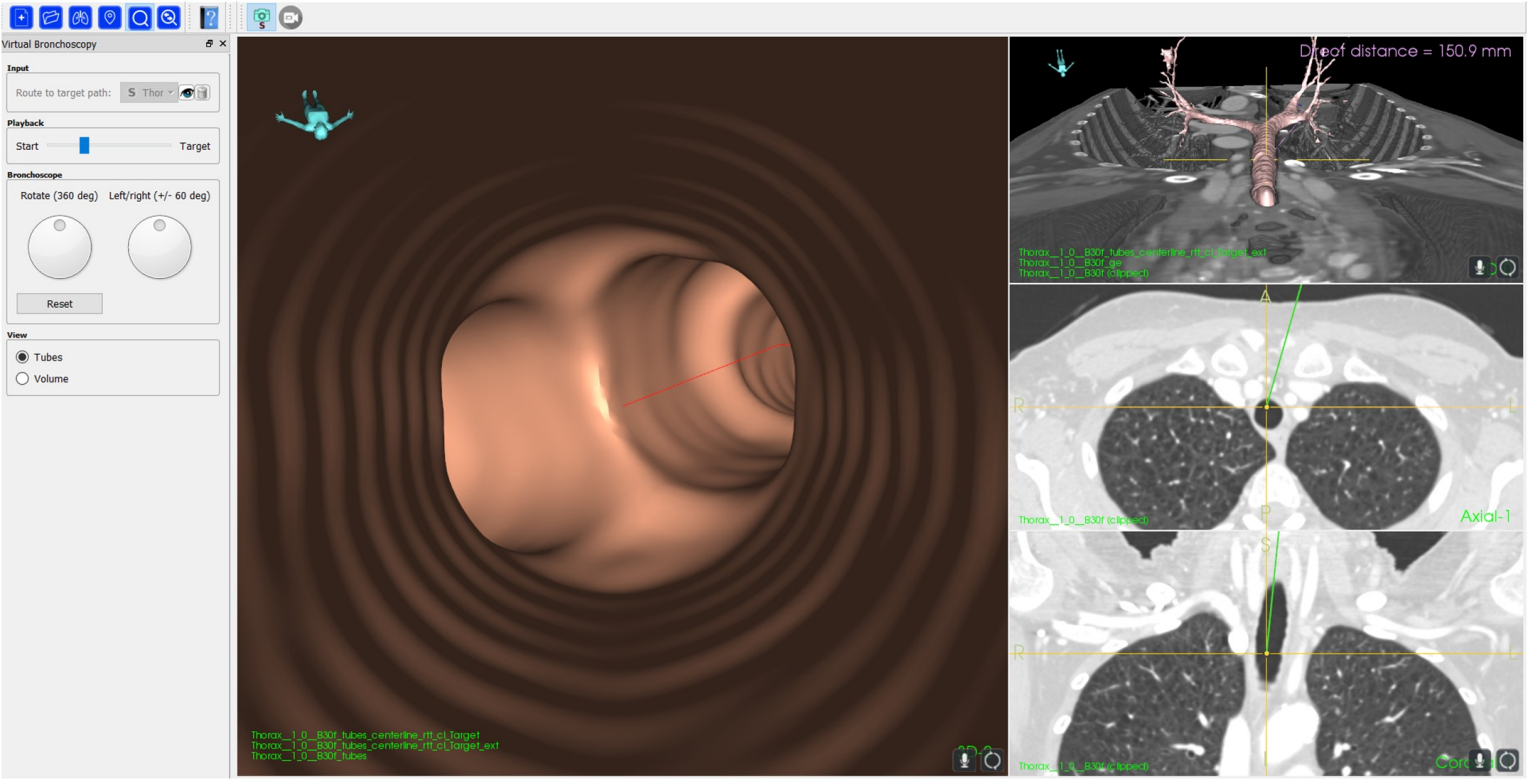
Screenshot of the VB *fly-through* mode from a positions along the route-to-target inside the airways.

**Fig 5:**
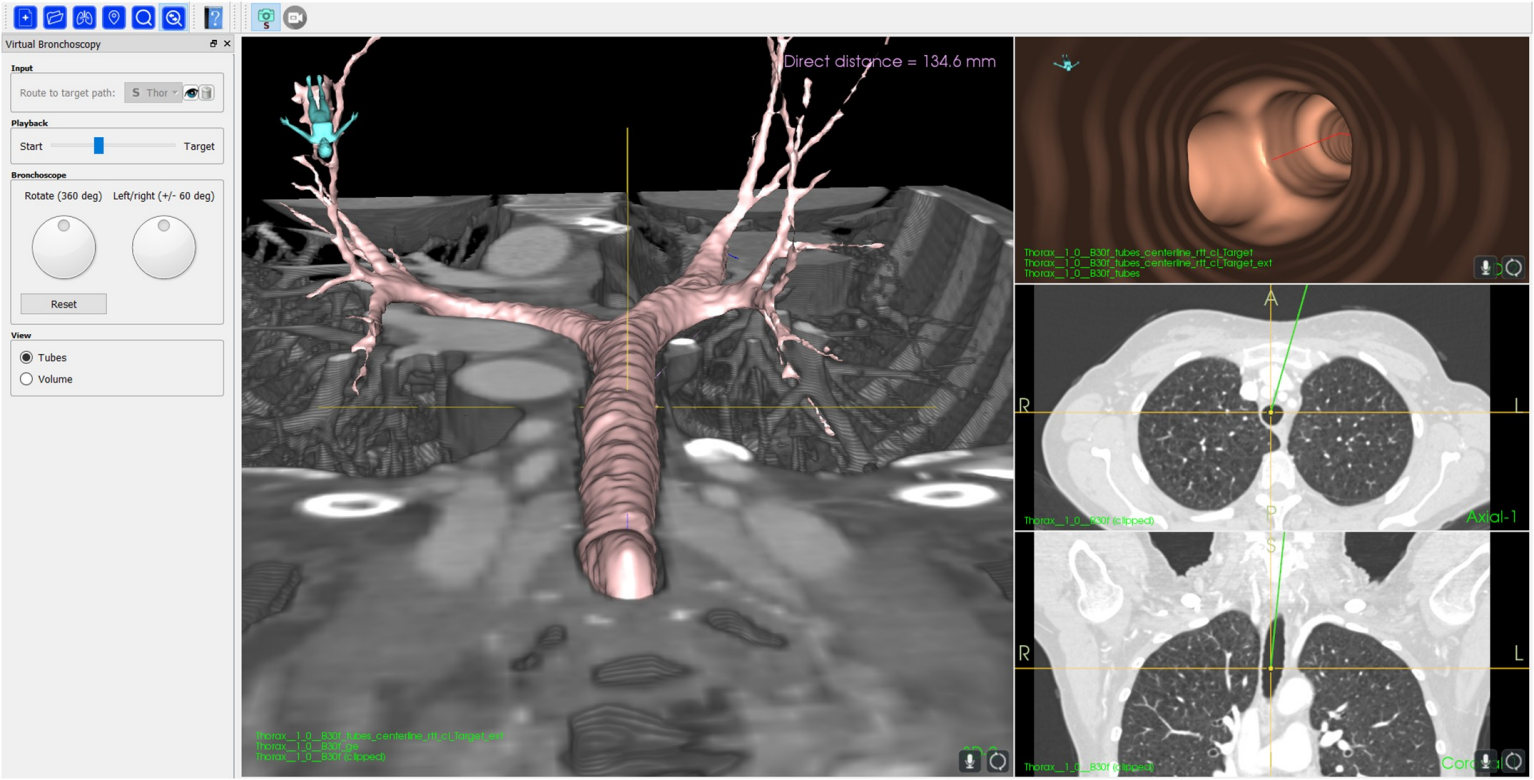
Screenshot of the VB *cut-plane* mode.

Two different VB modes were demonstrated in Fraxinus: A *fly-through* from inside the airways and the novel *cut-plane* mode, where the CT image is cut in the sagittal plane at the virtual position of the bronchoscope on the route-to-target. In the VB mode the main window shows the *fly-through* (Fig 4) or the *cut-plane* (Fig 5). In addition an overview of the 3D airways model and the axial and coronal CT planes at the virtual position of the bronchoscope is shown. A slider at the left of the window adjusts the virtual bronchoscope position. In addition, the view direction and rotation of the bronchoscope can be adjusted using a slider and a rotation wheel at the bottom left of the window. During virtual navigation the data was rendered at a sufficiently high speed with 20-25 frames per second (FPS), resulting in a smooth navigation with an instant response to any movement of the virtual camera.

## Related work

Fraxinus is not the only application developed on top of CustusX.

NorMIT (Norwegian Centre for minimally invasive image guided therapy and medical technologies) is a navigation platform for Image-Guided Therapy that is used as a national platform for IGT-related research in Norway. This navigation platform is based on the navigation systems CustusX and Hepa-Navi. Hepa-Navi is a planning and navigation tool for liver resection based on 3DSlicer, this system is referred to as NorMIT-Plan. The system based on CustusX, NorMIT-Nav (https://github.com/normit-nav), is built using the same approach described in this paper. The two parts of the NorMIT navigation platform are connected by a common library of algorithms, and data can be exchanged between the two systems using the OpenIGTLink protocol. The NorMIT-Nav specific code is a thin wrapper on top of CustusX, providing some simple GUI customizations.

The Fraxinus and NorMIT-Nav applications where developed by the same team that developed CustusX.

Navicad (Navigation system for confocal laser endomicroscopy to improve optical biopsy of peripheral lesions in the lungs) is a navigation platform for guiding flexible steerable catheters in the peripheral and central parts of the lungs (http://www.navicad.ro). The project platform was based on the CustusX platform for development. The planning part of Navicad is identical to Fraxinus as these parts both represents a solution to the same clinical challenge.

## Discussion

We have presented an open source software application, Fraxinus, which uses CustusX toolkit features to build a planning and guiding system for bronchoscopy. We have shown that it is possible to create a specialized application for this specific clinical procedure using CustusX.

### The size of the code base

The code base of Fraxinus is relatively small. Counting the number of source lines of code (SLOC) and number of commits in the Fraxinus and CustusX git repositories using the following commands (on a Mac) gives a indication on the amount of work needed to create a specialized application on top of CustusX:

git ls-files | xargs wc -l

git rev-list --all --count

The numbers are summarized in Table 1. The Fraxinus code base contains 1.7% SLOC compared to the CustusX code base and have had 1.5% of the number of commits.

**Table 1:** Number of source lines of code (SLOC) and number of commits in CustusX and Fraxinus.

	SLOC	Number of commits
CustusX	352894	14168
Fraxinus	6030	219

### Hiding vs. removing features

Fraxinus is tightly based on CustusX, the main window and most of the menus of both applications are in fact the same. Customizing the GUI is mainly done by modifying the workflow and loading/unloading plugins. This means that most features from CustusX are available in Fraxinus, but Fraxinus needs to present an uncluttered and user-friendly interface to the clinicians. Instead of removing, we are hiding (or not presenting) features which are not relevant in Fraxinus. There are several reasons for not removing features in this project.

The user interface is the top layer and most features are added deeper down in the code. Simply turning visibility off in the user interface rather than removing the feature is time saving during development. Some feature that we might have wanted to remove is tightly coupled with the rest of the system. The tool configuration in the preference menu is one such example.

Another possibility, that we decided not to to use in this project, is to keep the underlying features, and completely remove all user interfaces to these features. Using this method, it is possible to create an even simpler application, as the user would not be able to use the features at all.

Because the system is modularised, the existence of hidden features should not interfere with the desired functionality. The assumption here is that the hidden features are not resource consuming to a point where they compete with resources of the features of Fraxinus.

Keeping a full system core also makes it possible to implement more advanced navigation capabilities at a later stage in the Fraxinus project. The group has previously presented an advanced real-time navigation systems for bronchoscopy and endobronchial ultrasound (EBUS), using electromagnetic navigation based on CustusX [7, 8]. These features are available for advanced users.

### New project vs. injecting a plugin into the CustusX project

The usual way of creating a new CMake/C++ application is to create a new CMake project, add a new main.cpp file, create an executable and link in libraries from e.g. the CustusX platform.

When using the CustusX toolkit the process is slightly different. An application based on CustusX is technically a plugin and it resides inside the CustusX CMake project. To build this new application, which is added by the plugin, a CMake flag “CX_APP_Fraxinus” is enabled. At this point the CustusX application will still be built, but as we are not interested in it, we can disable it by setting “CX_APP_CustusX” to OFF. Libraries are linked to this application as normal.

This approach might be unfamiliar to developers, but it has some advantages. The fact that the application is injected into the CustusX superbuild system gives many features for free to the new application: the test framework is set up and working, there are scripts available for easily setting up a CI and the process of creating an installer is handled.

### Alternative platforms

Several open source projects are available as extensible platforms for developing image-guided applications. One of the goals of 3D Slicer [17] is to be a development platform for researchers within a clinical research environment and they have presented a way of using it as a platform for prototyping [18]. The work presented by Nardelli et al. [19] shows an example of 3D Slicer used to make an open-source airway segmentation platform. The Medical Imaging Interaction Toolkit (MITK) [11] states that it can be used, extended and deployed as needed, and that it provides modularization and extensibility on different levels. Both of these platforms use CTK plugins. Although 3D Slicer has the flexibility of plugins, the main user interface has been non-customizable until the addition of Slicelets (http://wiki.slicer.org/slicerWiki/index.php/Documentation/Nightly/Developers/Slicelets). Commercial toolkits like MeVisLab(http://www.mevislab.de) can also be used to create this kind of application, the difference being that the code is not open source [20].

### Future directions

During the planning and development of Fraxinus we identified points that can be improved, features which we did not have the resources to prioritize, and challenges.

*The code stability of Fraxinus* is dependent on the CustusX toolkit. CustusX is a mature research platform with a stable code base [6], but the introduction of the CTK/OSGi plugin framework is relatively new. With this came the addition of service interfaces for the plugins to implement, and CustusX underwent a rather considerable re-factoring when adapting the framework. CustusX also went open source, opening for third-party developers to extend the platform. These changes have put new demands on the platform compared to earlier when it was an in-house research system only. As the system adapts to these demands it is natural to expect change in the code base. This process presents opportunities for the community to influence the direction of change, but it also add the challenge of changing without breaking too many dependencies. Fraxinus will have to evolve as the CustusX platform settles, but it should be able to profit on the additions contributed directly into CustusX without much effort. When new features are available in CustusX, Fraxinus can easily customize its user interface as appropriate.

*Running on off-the-shelf computers* have been stated as one of the goals of Fraxinus, as the technology should be available without excessive hardware or software costs. Currently, some of the core algorithms of Fraxinus are hardware dependent. In particular, the processing unit and memory specifications currently require more than you would find in a cheap off-the-self computer. To achieve this goal we plan to optimize the algorithms, but we also anticipate that computers will become more powerful.

*DICOM import* is possible to automatize further by e.g. making assumptions regarding which data set is most relevant or importing several and presenting the (assumed) most relevant, but having others readily available for visualization.

*Intraoperative guidance* is possible using Fraxinus. Support for tracking systems is not made available in Fraxinus. Using the current Fraxinus interface for intraoperative guidance would require an assistant to control the VB simulation to match the position and orientation of the bronchoscope as the doctor performs the procedure. Future work can allow running the VB simulation in a loop, offering intraoperative guidance without an assistant or support for a cheap tracking system could be added.

*Positron emission tomography–computed tomography (PET-CT)* is an important image modality to clinicians, as it is a suitable tool for helping to identify tumors and malignancy. The current support for PET-CT is limited, but we hope to improve both the import possibilities and also the visualization of such type of medical images.

*Making Fraxinus usable for everyone* is a long-term goal of the project. Currently, Fraxinus can be used for research only, it is not FDA or CE-approved. Testing and validation for clinical use is the responsibility of the user. It can be used in the OR only after going through an ethics research board approval process. However, it is our intent to work towards this long-term goal to help improve treatment for everyone at a low cost.

## Conclusion

Fraxinus is a specialized image-guided planning application built on top of the open source image-guided intervention software platform, CustusX. The CustusX toolkit facilitated the development by offering extension points into many of the important steps of the software development process. Fraxinus is customized to the particular clinical needs in bronchoscopy. Addressing these needs have resulted in a tool which can offer free assistance to clinicians performing these procedures, currently within a research environment. The application is an example of an open source solution targeting a specific clinical need, that is freely available for further development and research. The software has the potential to become a useful tool for the future patient treatment.
